# Nomogram and Risk Calculator for Postoperative Tracheostomy after Heart Valve Surgery

**DOI:** 10.3390/jcdd10020073

**Published:** 2023-02-08

**Authors:** Xiangchao Ding, Bing Sun, Liang Liu, Yuan Lei, Yunshu Su

**Affiliations:** 1 Department of Thoracic Surgery, Renmin Hospital of Wuhan University, Wuhan 430064, China; 2 Department of Cardiovascular Surgery, Union Hospital, Tongji Medical College, Huazhong University of Science and Technology, Wuhan 430074, China; 3 Wuhan Third Hospital (Tongren Hospital of Wuhan University), Wuhan 430064, China; 4 Department of Cardiovascular Surgery, Renmin Hospital of Wuhan University, Wuhan 430064, China; 5 Department of Gerontology, Renmin Hospital of Wuhan University, Wuhan 430064, China

**Keywords:** tracheostomy, heart valve surgery, risk factor, nomogram, risk calculator

## Abstract

Postoperative tracheostomy (POT) is an important indicator of critical illness, associated with poorer prognoses and increased medical burdens. However, studies on POTs after heart valve surgery (HVS) have not been reported. The objectives of this study were first to identify the risk factors and develop a risk prediction model for POTs after HVS, and second to clarify the relationship between POTs and clinical outcomes. Consecutive adults undergoing HVS from January 2016 to December 2019 in a single cardiovascular center were enrolled, and a POT was performed in 1.8% of the included patients (68/3853). Compared to patients without POTs, the patients with POTs had higher rates of readmission to the ICU and in-hospital mortality, as well as longer ICU and hospital stays. Five factors were identified to be significantly associated with POTs after HVS by our multivariate analysis, including age, diabetes mellitus, pulmonary edema, intraoperative transfusion of red blood cells, and surgical types. A nomogram and a risk calculator were constructed based on the five factors, showing excellent discrimination, calibration, and clinical utility. Three risk intervals were defined as low-, medium-, and high-risk groups according to the nomogram and clinical practice. The findings of this study may be helpful for early risk assessment and perioperative management.

## 1. Introduction

Postoperative complications are prevalent after cardiovascular surgery, and a postoperative tracheostomy (POT) is often required to strengthen airway management when respiratory failure, circulatory failure, multiple organ dysfunction, and other critical adverse events develop [1,2,3]. As an important indicator of poor prognoses, POT operations often indicate a higher risk of mortality, prolonged hospital stay, increased medical burden, and declined quality of life [4,5,6,7]. The incidence of POTs varies widely in the previous literature due to the different surgical populations in different studies [3,7,8,9,10,11]. Compared to other surgical types, patients undergoing cardiovascular surgery have been reported to have a relatively higher POT rate, mostly in the range of 1.4–11.8% [3,12,13].

For patients who are expected to be unable to escape from mechanical ventilation in the short term, a tracheostomy is a routine operation to effectively relieve airway obstruction, reduce airway resistance, reduce airway dead space, and increase effective ventilation volume [10,14]. Previous studies have reported that among critical patients requiring prolonged mechanical ventilation in intensive care unit (ICU) settings, compared with patients who did not receive a tracheostomy, patients who received a tracheostomy had significantly declined mortality and improved prognoses, despite a longer duration of mechanical ventilation and hospitalization [14,15,16,17,18]. In some major operations, such as liver transplantation and heart surgery, patients requiring a tracheostomy have a significantly longer hospital stay and lower overall survival rate compared with patients who do not require a tracheostomy [4,10]. Several studies focused on POTs have been conducted in patients undergoing cardiovascular surgery due to the high prevalence and significantly poorer outcomes [3,5,10,19,20]. Some significant risk factors for POTs have been reported and several predictive systems have been established in previous studies [4,5,21,22]. However, relevant clinical studies conducted in this field are still currently limited, and none of these previous studies were carried out specifically in patients undergoing heart valve surgery (HVS). Therefore, our understanding of the risk factors for POTs after HVS needs to be deepened and the construction of a credible and convenient risk prediction model is still urgently needed.

The primary objective of this study was to identify independent risk factors, develop a risk prediction model for POTs after HVS, and perform risk stratification according to the established model. The second objective of this study was to clarify the relationship between POTs and in-hospital clinical outcomes, and thus to provide evidence-based support for clinical practice.

## 2. Methods

### 2.1. Ethical Statement

This study was conducted based on the Declaration of Helsinki’s ethical principles. The Ethics Committee of Union Hospital, Tongji Medical College, Huazhong University of Science and Technology approved this study. Due to its observational and retrospective nature, patients’ signed informed consent was not needed.

### 2.2. Study Population

This was a retrospective, observational single-center cohort study. From January 2016 to December 2019, consecutive adult patients undergoing HVS in our hospital were identified and analyzed. A number of conditions were excluded from the current study, including: (1) younger than 18 years; (2) immunodeficiency, immunosuppression, or organ transplant history; (3) intraoperative death, early postoperative death or discharge (within the first 48 h after surgery); and (4) incomplete data in medical records.

### 2.3. Data Collection

Clinical data were extracted from the hospital’s electronic medical record system, including preoperative, intraoperative, and postoperative variables. Preoperative data included demographics (gender, age, body mass index, smoking and drinking history), underlying conditions (hypertension, diabetes mellitus, chronic obstructive pulmonary disease, cerebrovascular disease, peripheral vascular disease, renal insufficiency, gastrointestinal tract disease, atrial fibrillation, pulmonary edema, New York Heart Association (NYHA) class, cardiac surgery history, and general surgery history), ultrasound results (pulmonary artery hypertension, pericardial effusion, left ventricular ejection fraction, diameters of the left atrium, left ventricle, right atrium, and right ventricle), and laboratory values (white blood cell count, red blood cell count, hemoglobin, platelet count, serum creatinine, serum albumin, and serum globulin). Intraoperative data included cardiopulmonary bypass time, aortic cross clamp time, surgical types (isolated valve surgery, combined coronary artery bypass grafting (CABG), and combined aortic surgery), and transfusion of red blood cells (RBCs). Postoperative data included the rates of readmission to ICU and in-hospital mortality, as well as the lengths of patients’ ICU and hospital stays.

### 2.4. Endpoints

The primary endpoint was tracheostomy operations after HVS in this study. All the operations were performed via a percutaneous route using disposable sterile percutaneous tracheostomy surgical instrument package at patients’ bedsides by experienced operators. In this study, tracheostomy was indicated for the following reasons: repeated intubation, predicted difficult reintubation, one or more failed trials of extubation, bypass of upper airway obstruction, prolonged mechanical ventilation, and tracheal access that was necessary for removing thick pulmonary secretions.

### 2.5. Statistical Analysis

Statistical analysis of the data was performed with IBM SPSS (version 26.0) and R software (version 4.0.5). Differences were considered to be statistically significant if the two-tailed *p* values were less than 0.05.

Categorical data were presented as numbers (proportions) and continuous variables were presented as means ± standard deviations or medians (interquartile ranges) according to whether they were normally distributed. For univariate analysis, categorical data were analyzed using chi-square test or Fisher’s exact test, and continuous variables were analyzed using Student’s *t*-test or Mann–Whitney U test, as appropriate. Factors initially screened by univariate analysis (*p* values less than 0.1) were then entered into a forward stepwise multivariate logistic regression procedure to identify significant risk factors for POTs after HVS. The results of multivariate analysis were presented as *p* values, coefficients, and odds ratios (ORs) with 95% confidence intervals (CIs). A nomogram and a web-based risk calculator were then constructed based on the multivariate logistic regression model. Finally, risk stratification was performed to further facilitate the clinical application based on the nomogram model.

The performance of the model was assessed with discrimination, calibration, and clinical utility. Bootstrap method with 1000 replicates was used for internal validation. The area under the receiver operating characteristic (ROC) curve (AUC) was used to assess the discrimination. The Hosmer–Lemeshow goodness-of-fit test and calibration plot were used to assess the calibration. The decision and clinical impact curves were used to assess the clinical utility.

## 3. Results

### 3.1. Demographic Characteristics

A total of 3853 adult patients undergoing HVS met the inclusion criteria and were analyzed in the current study. The average age of these patients was 51.3 ± 12.5 years. Female patients accounted for 46.2% of the patients. The incidence rate for POTs in this population was 1.8% (68/3853).

The average body mass index of this study population was 23.0 ± 3.3 kg/m^2^. A total of 20.1% of the patients had a history of drinking and 26.7% had a history of smoking. A significant proportion of patients had at least one underlying disease, including pulmonary artery hypertension (32.1%), general surgery history (29.7%), hypertension (24.2%), atrial fibrillation (23.3%), pericardial effusion (15.6%), chronic obstructive pulmonary disease (12.9%), renal insufficiency (8.2%), gastrointestinal tract disease (8.2%), cardiac surgery history (8.0%), pulmonary edema (6.0%), and diabetes mellitus (5.7%).

Isolated valve surgeries were performed on 75.2% of the patients, combined CABG on 12.5%, and combined aortic surgeries on 12.3%. The median cardiopulmonary bypass time was 108 (86, 139) minutes, the aortic cross clamp time was 72 (54, 95) minutes, and the transfusion of intraoperative RBC was 1 (1, 3) units, respectively. The incidence rate for POTs was 0.9% in the patients undergoing isolated valve surgeries, 3.7% in those undergoing concomitant CABG, and 5.1% in those undergoing concomitant aortic surgeries.

The types of HVS are as follows: isolated aortic valve surgeries accounted for 26.8%, isolated mitral valve surgeries 25.1%, isolated tricuspid valve surgeries 8.1%, combined aortic and mitral valve surgeries 15.8%, combined aortic and tricuspid valve surgeries 1.2%, combined mitral and tricuspid valve surgeryies 15.8%, and combined aortic, mitral, and tricuspid valve surgeries 7.2%. Their corresponding POT rates were, respectively, 2.4%, 1.2%, 1.0%, 1.6%, 2.2%, 2.1%, and 1.1%.

### 3.2. Development of the Nomogram and Risk Calculator

A univariate analysis was first conducted to explore possible risk factors for POTs after HVS (Table 1).

The factors screened (*p* values less than 0.1) by the univariate analysis were then entered into a forward stepwise multivariate logistic regression procedure to further identify independent risk factors, including gender, age, smoking history, hypertension, diabetes mellitus, chronic obstructive pulmonary disease, cerebrovascular disease, renal insufficiency, pulmonary edema, cardiac surgery history, NYHA class, white blood cell count, red blood cell count, platelet count, serum creatinine, serum albumin, surgical types, cardiopulmonary bypass, and intraoperative transfusion of RBCs. A multicollinearity test was conducted before the regression analysis in order to exclude confounded variables with potential multicollinearity. Finally, five independent risk factors for POT after HVS were identified in the multivariate logistic regression analysis, including being of an older age, diabetes mellitus, pulmonary edema, combined aortic surgeries, and more transfusions of intraoperative RBCs (Table 2).

**Figure 1:**
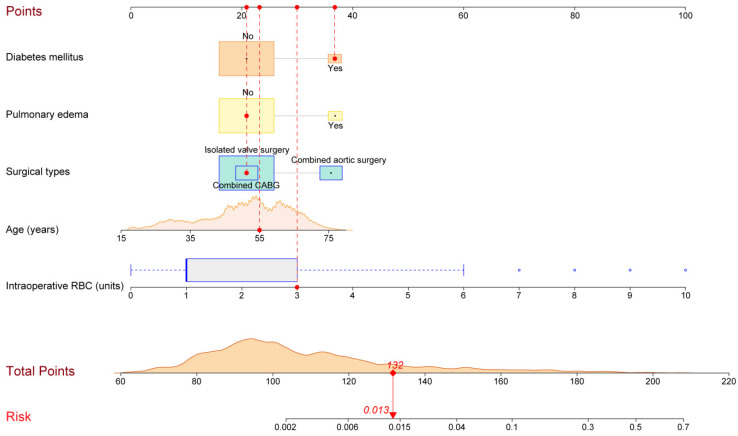
Nomogram for the prediction of postoperative tracheostomy after heart valve surgery. A specific patient was presented to show the usage of the nomogram. This was a 55-year-old patient with diabetes mellitus and without pulmonary edema. He underwent isolated valve surgery and was transfused with 3 units of RBCs intraoperatively. The individual item points corresponding to each variable are shown at the top, and the total scores are obtained from the sum of the points corresponding to each variable by a red dot. Given values of the five variables, the patient can be intuitively mapped onto the nomogram. It can be clearly seen from the nomogram that the total score of this patient was 132 points and the corresponding probability of POT was 0.013. RBC, red blood cell. The squares of each color represent a single variable: orange squares indicate diabetes mellitus, yellow squares indicate pulmonary edema, and blue squares indicate surgical types.

Based on the logistic regression model established using the above five risk factors, we constructed a graphical nomogram for convenience in clinical use (Figure 1). The nomogram can proportionally convert each regression coefficient in the multivariate analysis to a scale of 0–100 points, reflecting their relative importance. The individualized POT risk of each patient can be easily obtained by summing the points of all the five risk factors and then identifying the corresponding probability at the bottom of the nomogram. Older patients who have had diabetes mellitus, pulmonary edema, combined aortic surgeries, and more intraoperative transfusions of RBCs may obtain more points and thus are at a higher risk of POTs after HVS. An example showing the usage of the nomogram is given in Figure 1.

**Figure 2:**
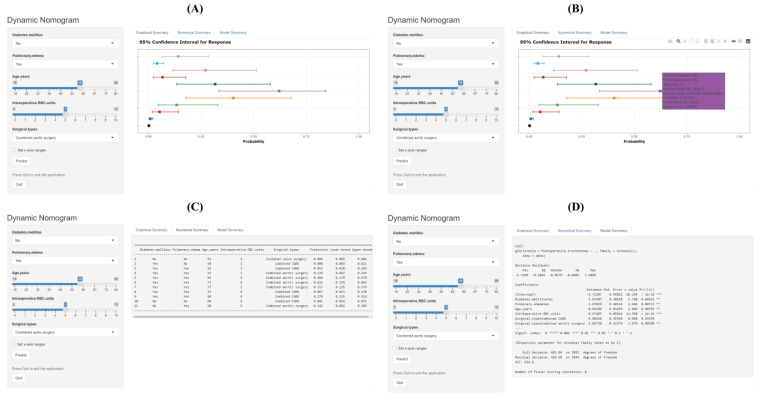
The usage of the internet-based risk calculator for POTs after HVS. (**A**) The estimated probabilities and corresponding 95% CIs of POT of 11 different patients are presented. The squares represent the estimates and the bars reflect their 95% CIs. (**B**) The detailed information of a patient and the corresponding estimated risk with 95% CI will appear when the square is clicked. (**C**) All the information of the patients can be acquired by clicking the “Numerical Summary”. (**D**) The information of the model can be acquired by clicking the “Model Summary”. CI, confidence interval; HVS, heart valve surgery; RBC, red blood cell; POT, postoperative tracheostomy. The lines of each color represent the risk in one case.

To facilitate the usage of this system in modern clinical work, we further created an internet-based risk calculator (available at https://pothvs.shinyapps.io/dynnomapp/, accessed on 7 December 2022). When using this online predictive system, we only need to choose the information of the patient and then click the “Predict” button; the estimated risk of POTs after HVS is calculated in the “Graphical summary” area on the right (Figure 2). When calculating the risk of another patient, one can simply change the information on the left. This makes it possible to assess the risks of multiple patients simultaneously, as well as the risk comparison among different patients. The specific information of the patients and the model can also be obtained by clicking the “Numerical summary” and “Model summary” on the right. When a user cannot log in with a new device, we recommend logging out first by pressing the “Quit” button at the left bottom and then reloading the procedure.

### 3.3. Validation and Assessment of the Model

**Figure 3:**
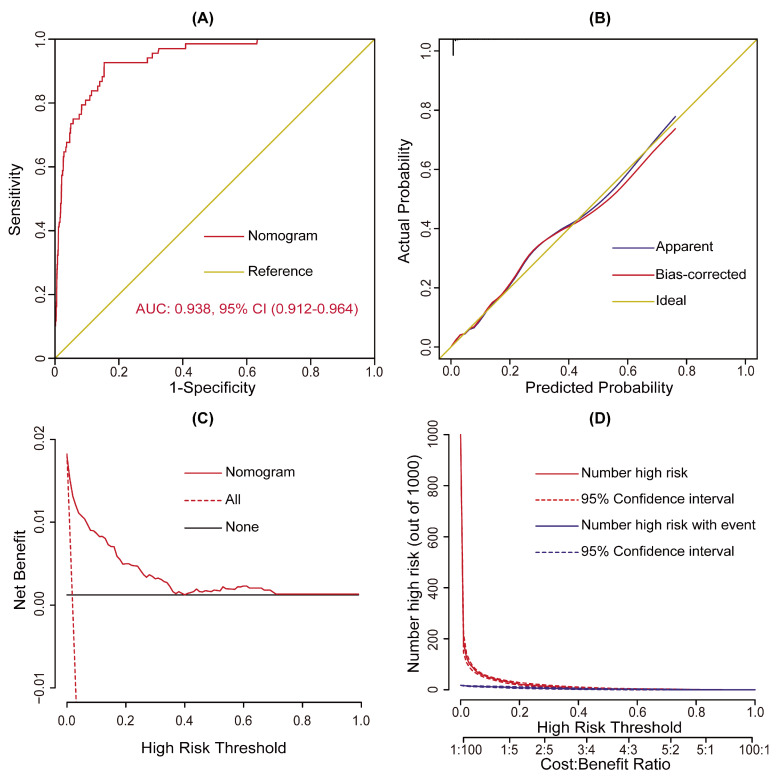
Assessment of the risk prediction model for postoperative tracheostomy after heart valve surgery. (**A**) ROC curve and the corresponding AUC, (**B**) calibration curves, (**C**) decision curve, and (**D**) clinical impact curve of the model. AUC, area under the receiver operating characteristic curve; CI, confidence interval; ROC, receiver operating characteristic curve.

The model was well validated internally by the bootstrap method with 1000 replicates. By plotting the ROC curves and calculating the AUC, the model demonstrated excellent discrimination, with an AUC of 0.938 (95% CI, (0.912–0.964), Figure 3A). By plotting the calibration curves and the goodness-of-fit test, the model demonstrated good consistency between the predicted and the actual probabilities, with a Hosmer–Lemershow chi-square value of 3.260 (*p* = 0.860, Figure 3B). By plotting the decision and clinical impact curves, the model demonstrated good clinical utility (Figure 3C,D), which may bring more clinical net benefits compared to the “treat-all/none” strategies.

### 3.4. Risk Stratification

To facilitate clinical applications, we further propose a more concise risk stratification for POTs after HVS on the basis of the nomogram and clinical practice (Table 3).

**Figure 4:**
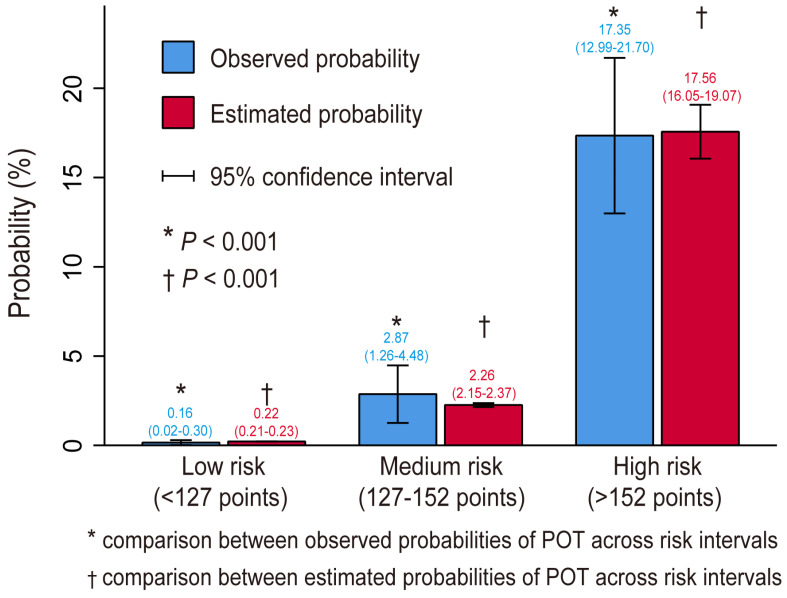
Bar chart showing the consistency between the observed and estimated probabilities. The difference between the observed and estimated probabilities in the same risk interval was not significant (*p* > 0.05) but the difference was significant between different risk intervals (*p* < 0.05), demonstrating good consistency and reasonable division.

We stratified all the patients into three risk intervals: low-risk, medium-risk, and high-risk groups. The selected cutoffs of the estimated probabilities were, respectively, 0.01 and 0.05, corresponding to scores of 127 and152 points on the nomogram. In the current study, 81.5% of the patients were stratified into the low-risk group, 10.9% into the medium-risk group, and 7.6% into the high-risk group. Both the estimated and observed probabilities demonstrated a significant difference across the three risk intervals and the estimated and observed probabilities showed good consistency within each risk interval, indicating the rationality of the risk stratification (Figure 4).

### 3.5. Clinical Outcomes

The overall mortality rate was 2.9% (111/3853) in the current study, with a significant increase in patients who experienced POTs (57.4% versus 1.9%, *p* < 0.001). In addition, the rate of readmission to ICU was significantly higher in patients with POTs, and the lengths of their ICU and hospital stays were significantly longer compared to those of patients who did not require POTs. The comparison details of these outcomes between patients with and without POTs are presented in Table 4.

For the 68 patients who underwent POTs after HVS, five patients were operated on within the first postoperative week, with a mortality rate of 20.0% (1/5); forty-one patients were operated on between the first and the second postoperative week, with a mortality rate of 58.5% (24/41); and twenty-two patients were operated on after two postoperative weeks, with a mortality rate of 63.6% (14/22).

## 4. Discussion

Undergoing a tracheostomy is an important indicator of the increased risk of poor prognoses in patients undergoing cardiovascular surgeries [3,7,9,20], which was again confirmed in the current study. Due to the difference of surgical populations in different studies, the reported rates of POTs in the previous literature were quite different [3,7,8,9,10,11]. The overall incidence rate of POTs after HVS was 1.8% in our analysis, falling within the range of incidence rates reported in the previous literature [3,12,13]. The overall mortality rate was 2.9%; however, patients with POTs had a significantly higher rate of mortality compared with patients without POTs. Moreover, a higher rate of readmission to the ICU, as well as prolonged ICU and hospital stays were also observed in patients with POTs. The increased risk of multiple poor clinical outcomes in patients with POTs stressed the importance of identifying significant risk factors for POTs after HVS and developing a compelling risk prediction model.

In the current study, using clinical data of 3853 adult patients who underwent HVS at a single cardiovascular center, we analyzed the risk factors of POTs after HVS and developed a parsimonious risk prediction model. Through a univariate and multivariate analysis, we identified five independent risk factors for POT after HVS, including being of an older age, having diabetes mellitus, pulmonary edema, combined aortic surgery, and more transfusions of intraoperative RBCs. To facilitate the clinical application of the logistic regression model, we further constructed a visual nomogram and an internet-based risk calculator. The model demonstrated excellent discrimination, calibration, and clinical utility, and was well validated internally. On the basis of the nomogram and clinical practice, we defined three risk intervals: low-risk, medium-risk, and high-risk groups. To the best of our knowledge, this is the first report that has targeted the risk factors of POTs after HVS and the first attempt to construct a nomogram model and an internet-based risk calculator worldwide, which may have certain clinical guiding significance.

Numerous studies have been conducted on POTs after various surgical procedures due to the adverse outcomes, and the risk factors identified in our analysis have also been reported in different reports [3,4,5,19,20,21,22]. Being of an older age was identified to associate with a higher risk of POTs in the current study; however, the results of whether the risk of POTs would increase with age were inconsistent in previous studies, which may be due to the difference in disease types and study populations [4,22,23]. Diabetes mellitus and pulmonary edema as risk factors for POTs have also been reported in previous studies, which may be mainly associated with higher risks of various pulmonary complications [7,24,25]. The relationship between combined aortic surgery and ventilation dependence was reported a long time ago, and patients undergoing aortic procedures have been identified to have a higher risk of respiratory failure [26,27]. Intraoperative transfusions of RBCs may significantly increase the risk of transfusion-related acute lung injury and systemic inflammatory response syndrome, which may lead to prolonged hospitalization, increased medical costs, and a higher risk of mortality [28,29,30]. Although RBC transfusions are routine in traditional cardiovascular surgery to deal with bleeding and improve tissue oxygen delivery, there is growing evidence that the restrictive RBC transfusion strategy is safe and effective, which has been recommended by practice guidelines [31,32,33,34]. In addition, previous studies have found that the risk factors identified in this study are related to the development of various postoperative respiratory complications, such as pneumonia to some extent, which may indirectly increase the risk of the need for POTs [35,36,37,38,39,40,41,42].

Several other factors have also been reported to be related to an increased risk of POTs in previous studies but were not identified in the current study, including renal insufficiency, chronic obstructive pulmonary disease, white blood cell count, smoking history, body mass index, and platelet transfusion [4,5,7,9,43]. Additionally, although some postoperative variables have been identified to be related to POTs in the literature [44], we did not include these variables in our analysis. This was because the inclusion of these variables would not achieve the purpose of early prediction as they were not available early. Nonetheless, the results of our analysis demonstrated that a model constructed using only the preoperative and intraoperative variables identified in this study could also perform well.

Using the nomogram and risk calculator, we can accurately and easily estimate personalized POT risks, identify high-risk subsets and then take early and appropriate intervention measures. In the past few years, some measures have been proposed to be effective in reducing the risk of POTs, such as prophylactic administration of sivelestat at the beginning of cardiopulmonary bypasses. Taking appropriate measures targeting high-risk patients identified by our risk prediction model may significantly improve prognoses and achieve greater financial success.

Tracheostomies have been proven to be an effective treatment for various critically ill patients in recent years [15,17,45]. For patients undergoing high-risk surgeries, such as cardiovascular procedures, performing tracheostomies at an optimal time point when needed may significantly improve their prognoses [3,8,19,20]. However, the optimal timing is still unclear and controversial even though a lot of effort has been made by scientists and clinicians [1,8,16]. The results of this study showed that the in-hospital mortality rate increased in patients undergoing late POTs compared to patients undergoing early POT, consistent with the majority of the published reports [3,8,11,16,20]. However, we cannot simply conclude that the earlier the POT is performed, the better the prognosis will be. We must realize that this result was only based on a small sample which only included 68 patients who underwent POTs, and we cannot guarantee that all these patients had the same basic conditions when the POTs were performed, which may also have a significant impact on patients’ outcomes. Therefore, a prospective large sample study is still needed to further determine the timing of POTs after cardiovascular surgery.

There are several limitations in the current study that should be noted. First, this was a single-center study and was not validated externally in an independent dataset, which may limit the generalizability of our findings. Second, some possible risk factors, such as the N-terminal fragment of B-type natriuretic propeptide [46], were not collected and included in our analysis, even though the established model performed well. Third, the data we collected were limited to hospitalization, and long-term prognoses after discharge were not followed or analyzed, which needs to be strengthened in future studies. Fourth, due to the nature of the retrospective observational real-world study, we cannot accurately judge whether there were some patients who needed POTs theoretically but POTs were not performed actually among the dead patients, and therefore we did not know whether a POT would reduce the expected mortality in this subset of the patients, which may lead to some difference between the actual situation and the ideal judgment.

## 5. Conclusions

A POT after HVS is not uncommon, and is associated with poorer outcomes. In a multivariate analysis, five independent risk factors for POTs after HVS were identified, including being of an older age, having diabetes mellitus, pulmonary edema, combined aortic surgery, and more transfusions of intraoperative RBCs. The prediction model constructed using the above five risk factors demonstrated excellent discrimination, calibration, and clinical utility, which may be helpful for early risk assessment and perioperative management. A graphical nomogram and an internet-based risk calculator were constructed to facilitate clinical applications on the basis of the multivariate model, and three risk groups were defined based on the nomogram and clinical practice. To our knowledge, this is the first report that has targeted the risk factors of POTs after HVS and the first attempt to construct a nomogram model and an internet-based risk calculator worldwide, which may have certain clinical guiding significance.

## Figures and Tables

**Table 1:** Univariate analysis of possible risk factors for POTs after HVS.

Characteristic	Without POT n = 3785 (%)	With POT n = 68 (%)	χ^2^/Z/t	*p* Value
Demographics				
Male	2028 (53.6)	45 (66.2)	4.264	0.039
Age (years)	51.12 ± 12.53	58.82 ± 11.10	5.035	<0.001
Body mass index (kg/m^2^)	23.02 ± 3.26	23.19 ± 4.16	0.323	0.748
Smoking history	1003 (26.5)	26 (38.2)	4.700	0.030
Drinking history	759 (20.1)	14 (20.6)	0.012	0.913
Underlying conditions				
Hypertension	899 (23.8)	34 (50.0)	25.079	<0.001
Diabetes mellitus	209 (5.5)	11 (16.2)	14.085	<0.001
Chronic obstructive pulmonary disease	481 (12.7)	17 (25.0)	8.968	0.003
Cerebrovascular disease	1325 (35.0)	36 (52.9)	9.405	0.002
Peripheral vascular disease	1582 (41.8)	34 (50.0)	1.846	0.174
Renal insufficiency	296 (7.8)	19 (27.9)	36.024	<0.001
Gastrointestinal tract disease	307 (8.1)	9 (13.2)	2.330	0.127
Atrial fibrillation	882 (23.3)	14 (20.6)	0.276	0.599
Pulmonary edema	221 (5.8)	11 (16.2)	12.615	<0.001
Cardiac surgery history	299 (7.9)	11 (16.2)	6.185	0.013
General surgery history	1124 (29.7)	19 (27.9)	0.099	0.754
NYHA class III–IV	679 (17.9)	18 (26.5)	3.281	0.070
Pulmonary artery hypertension	1218 (32.2)	19 (27.9)	0.551	0.458
Pericardial effusion	587 (15.5)	15 (22.1)	2.174	0.140
Diameter of the left atrium (cm)	4.5 (3.9, 5.3)	4.6 (3.9, 5.1)	0.337	0.736
Diameter of the left ventricle (cm)	5.3 (4.6, 6.0)	5.0 (4.6, 5.8)	0.563	0.574
Diameter of the right atrium (cm)	3.9 (3.5, 4.5)	4.1 (3.6, 4.9)	1.635	0.102
Diameter of the right ventricle (cm)	3.6 (3.3, 4.0)	3.7 (3.4, 4.4)	1.557	0.120
Left ventricular ejection fraction (%)	62 (58, 66)	62 (57, 66)	0.144	0.866
Laboratory values				
White blood cell count (×109/L)	5.6 (4.7, 6.8)	6.0 (4.9, 8.7)	2.307	0.021
Red blood cell count (×1012/L)	4.3 (3.9, 4.6)	4.1 (3.7, 4.7)	1.787	0.074
Hemoglobin (g/L)	129 (118, 141)	124 (114, 141)	1.216	0.224
Platelet count (×109/L)	176 (142, 215)	152 (107, 209)	2.677	0.007
Serum creatinine (μmol/L)	71.7 (61.0, 84.4)	80.6 (66.6, 101.6)	3.701	<0.001
Serum albumin (g/L)	40.5 (38.0, 42.7)	39.3 (36.7, 41.7)	2.824	0.005
Serum globulin (g/L)	24.3 (21.6, 27.1)	24.9 (21.2, 29.5)	0.803	0.422
Operative variables				
Surgical types			53.035	<0.001
Isolated valve surgery	2871 (75.9)	26 (38.2)		
Combined CABG	463 (12.2)	18 (26.5)		
Combined aortic surgery	451 (11.9)	24 (35.3)		
Cardiopulmonary bypass time (minutes)	108 (85, 138)	153 (105, 236)	5.128	<0.001
Aortic cross clamp time (minutes)	72 (53, 95)	92 (58, 137)	4.003	<0.001
Transfusion of red blood cells (units)	1 (1, 3)	8 (5, 9)	12.326	<0.001

CABG, coronary artery bypass grafting; HVS, heart valve surgery; NYHA, New York Heart Association; POT, postoperative tracheotomy.

**Table 2:** Multivariate analysis of independent risk factors for POTs after HVS.

Characteristic	Coefficient	Standard Error	OR (95% CI)	*p* Value
Age (years)	0.042	0.015	1.043 (1.014–1.073)	0.004
Diabetes mellitus	1.074	0.385	2.927 (1.376–6.229)	0.005
Pulmonary edema	1.078	0.401	2.940 (1.338–6.457)	0.007
Transfusion of red blood cell (units)	0.675	0.058	1.964 (1.752–2.201)	<0.001
Surgical types				0.004
Isolated valve surgery	Reference	Reference	Reference	Reference
Combined CABG	0.003	0.356	1.003 (0.499–2.013)	0.994
Combined aortic surgery	1.028	0.334	2.794 (1.453–5.375)	0.002
Intercept	−9.722	0.948	<0.001	<0.001

HVS, heart valve surgery; CI, confidence interval; OR, odds ratio; POT, postoperative tracheotomy.

**Table 3:** Risk intervals of POT based on the nomogram.

Risk Intervals	Low Risk (<127 Points)	Medium Risk (127–152 Points)	High Risk (>152 Points)
Estimated probability (%)	<1	1–5	>5
Observed probability, % (95% CI)	0.16 (0.02–0.30)	2.87 (1.26–4.48)	17.35 (12.99–21.70)
No. of patients (%)	3141 (81.5)	418 (10.9)	294 (7.6)

Abbreviations: CI, confidence interval; POT, postoperative tracheotomy.

**Table 4:** Postoperative variables in patients with and without POTs after HVS.

Variables	Without POT n = 3785 (%)	With POT n = 68 (%)	χ^2^/Z	*p* Value
Readmission to ICU	119 (3.1)	26 (38.2)	227.125	<0.001
ICU stay (days)	2.8 (1.9, 3.9)	18.8 (11.4, 27.1)	12.611	<0.001
Hospital stay (days)	14 (11, 19)	37 (25, 48)	11.839	<0.001
Mortality	72 (1.9)	39 (57.4)	734.110	<0.001

HVS, heart valve surgery; ICU, intensive care unit; POT, postoperative tracheotomy.

## Data Availability

The data presented in this study are available on request from the corresponding author.

## Abbreviations

AUC	area under the receiver operating characteristic curve
CABG	coronary artery bypass grafting
CI	confidence interval
HVS	heart valve surgery
ICU	intensive care unit
NYHA	New York Heart Association
OR	odds ratio
RBC	red blood cell
ROC	receiver operating characteristic curve
POT	postoperative tracheostomy
